# A putative rRNA methyltransferase Mrm1 regulates mitochondrial dynamics and pathogenicity in *Magnaporthe oryzae*

**DOI:** 10.1080/21505594.2026.2715237

**Published:** 2026-08-12

**Authors:** Mi Shen, Xiaowen Xu, Shikun Xiang, Xiao-Lin Chen

**Affiliations:** a Hubei Zhongke Research Institute of Industrial Technology, College of Biology and Agricultural Resources, Huanggang Normal University, Huanggang, China; b Hubei Academy of Forestry, Institute of Forest Protection, Wuhan, China; c State Key Laboratory of Agricultural Microbiology and Provincial Key Laboratory of Plant Pathology of Hubei Province, College of Plant Science and Technology, Huazhong Agricultural University, Huanggang, China

**Keywords:** *Magnaporthe oryzae*, Mrm1, rRNA methylation, mitochondrial dynamics, pathogenicity, rice blast disease

## Abstract

Rice blast disease, a major global threat to staple crops, is caused by the ascomycete fungus *Magnaporthe oryzae*. This pathogen has complex mechanisms to invade rice, with mitochondrial function crucial for infection energy. Our study looks at the impact of Mrm1, a putative rRNA methyltransferase, on mitochondrial dynamics and pathogenicity of *M. oryzae*. Mrm1 deficiency delays appressorium formation and reduces turgor pressure for host penetration and infection hypha expansion. The N-terminal sequence of Mrm1, with a mitochondrial targeting sequence (MTS), is vital for its localization and function. Deletions cause impaired growth and lower pathogenicity. Deleting *MRM1* leads to abnormal mitochondrial morphology, with more filamentous mitochondria during invasive growth, disrupting the balance of fission and fusion. This imbalance reduces the fungus’s infection ability. Furthermore, loss of Mrm1 alters the steady-state protein levels of mitochondrial dynamics regulators Dnm1 and Fzo1, likely through translational regulation, while their transcript abundances remain unchanged. In the absence of Mrm1, the levels of these proteins are significantly reduced. Our findings deepen the understanding of epitranscriptomic regulation in fungal pathogenicity and represent a potential candidate for future target-based intervention strategies, pending validation through chemical or genetic approaches.

## Introduction

Rice blast, caused by *Magnaporthe oryzae*, ranks among the most destructive diseases threatening global rice production [1]. This ascomycete pathogen is caused by the ascomycete fungus *Magnaporthe oryzae*, which has evolved intricate mechanisms to infect rice plants. The infection process of this pathogen involves a series of well-coordinated steps, starting with conidial germination and ending with the formation of appressoria – the forceful penetration pegs that breach the host’s cuticle and facilitate invasion [2,3].

During the process of appressorium formation, the cytoskeleton undergoes reorganization, with microtubules and microfilaments playing crucial roles in shaping the appressorium. Lipid droplets accumulate and supply energy and structural precursors for the development of the appressorium. Mitochondria also shift in number, distribution, and function to meet the increased energy demands. These physiological, biochemical, and subcellular structural changes work together to ensure the successful formation and functionality of the appressorium in the infection process [3,4].

Recent research has highlighted the importance of mitochondrial dynamics for the pathogenicity of *M. oryzae* [5–7]. The balance between mitochondrial fission and fusion is essential for the adaptation and survival of this pathogen within host tissues [5]. A key player in this balance is the mitochondrial outer membrane translocase MoTom20, which modulates mitochondrial morphology and is crucial for the infectious growth of *M. oryzae* [6]. Disruption of genes involved in mitochondrial fusion, such as *MoFZO1*, or fission, such as *MoDNM1*, leads to significant reductions in pathogenicity, indicating the importance of a filamentous-punctate-filamentous cycle in mitochondrial morphology during the *Magnaporthe*-rice interaction [7]. Moreover, the availability of nutrients has been shown to regulate mitochondrial dynamics, with exogenous carbon sources delaying the transition in mitochondrial dynamics during invasive growth. In contrast, carbon starvation induces the breakdown of the mitochondrial network, leading to more punctate mitochondria in vitro. This nutrient-based regulation precedes mitophagy, a process mediated by MoAtg24, which is essential for proper biotrophic development and invasive growth in planta [5]. The findings underscore the significance of mitochondrial dynamics and mitophagy in the transition from biotrophy to necrotrophy and are necessary for the proper induction and establishment of blast disease in rice. Characterizing these mechanisms could provide valuable insights into developing strategies to control rice blast disease by targeting the pathogen’s ability to modulate its mitochondrial dynamics in response to host cues.

Epitranscriptomic RNA modifications, including 2”-O-methylation of rRNA, constitute a widespread yet complex research field. Studies across species from bacteria to humans link rRNA 2‘-O-methylation to ribosomal maturation and stability, which further modulate protein synthesis and cellular function [8–10]. This modification stabilizes rRNA and fine-tunes translation efficiency [10]. Mechanistically, small nucleolar RNAs (snoRNAs) guide methylation by complementary base pairing with specific rRNA sequences to ensure site-specific modification [11]. Recent studies have also highlighted the significance of 2’-O-methylation in the context of gene expression regulation. For instance, internal 2‘-O-methylation of mRNA has been shown to serve as a regulatory mechanism controlling mRNA abundance and protein levels *in vivo* [11,12]. This marks 2’-O-methylation as a pervasive post-transcriptional regulatory mechanism with broad cellular impacts and relevance to human diseases such as cancer [13]. Collectively, rRNA 2”-O-methylation catalyzed by standalone methyltransferases shapes RNA stability and translational control across kingdoms. Conserved Mrm1 methyltransferases from yeast to humans mediate this rRNA modification, reflecting evolutionary conservation of this epitranscriptomic pathway.

The study of RNA modifications in plant pathogenic fungi, including *M. oryzae*, is an emerging field. Recent advances have emphasized the significance of these modifications in regulating fungal development and pathogenicity. Modifications such as m^6^A in mRNA have been shown to influence gene expression in fungi like *M. oryzae*, affecting virulence factors and stress responses [14]. However, Mrm1-mediated rRNA 2’-O-methylation remains largely uncharacterized in phytopathogenic fungi, especially *M. oryzae*, creating a critical gap in our understanding of fungal epitranscriptomic regulation.

Drawing on established knowledge of rRNA 2’-O-methylation and its translational impacts, this study focuses on the role of Mrm1 in *M. oryzae*. We reveal a direct link between Mrm1-dependnt rRNA methylation and the control of mitochondrial fission and fusion during pathogen infection in *M. oryzae*. We demonstrate that the deletion of *MRM1* leads to abnormal mitochondrial morphology and attenuates pathogenicity, confirming Mrm1 as a central modulator of fungal virulence. Our findings advance the mechanistic understanding of RNA modification-mediated fungal pathogenicity and identify Mrm1 as a candidate target for novel rice blast control approaches.

## Results

### Evolutionary conservation and functional domains of Mrm1 in M. oryzae

**Figure 1.**
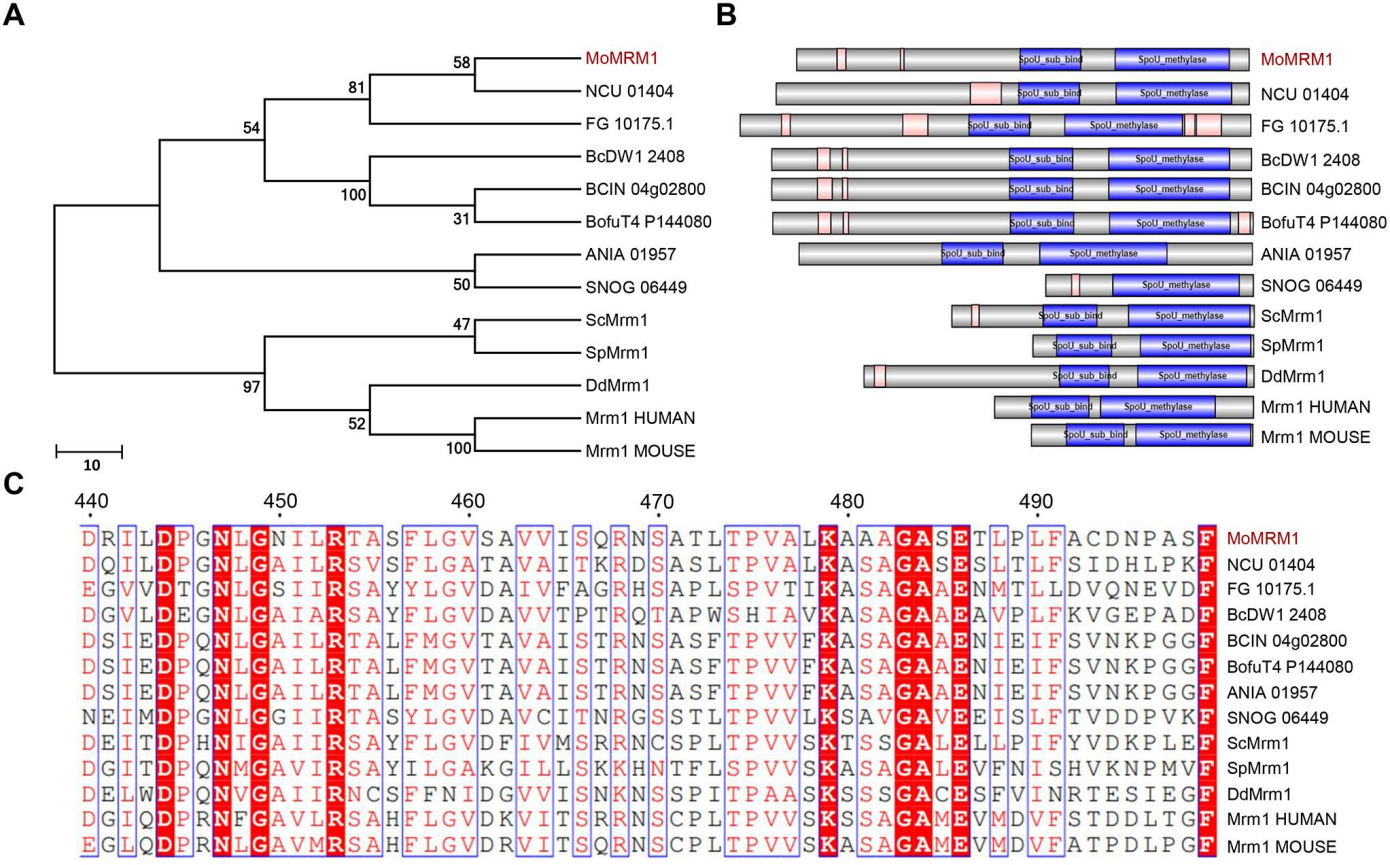
Comparative analysis of Mrm1 protein domains and phylogenetic relationships across species.

The putative rRNA methyltransferase Mrm1 was initially identified in mammals, including humans (*Homo sapiens*) and mice (*Mus musculus*). A homolog of this gene, also annotated as Mrm1 (MGG_00735), exists in the rice blast fungus. Homology searches and comparisons within the UniProt database revealed that the *M. oryzae* Mrm1 shares the closest phylogenetic relationship with its *Neurospora crassa* counterpart (Figure 1(A)). Structural analysis using the SMART database predicted the presence of both a substrate-binding domain and a methyltransferase catalytic domain, which are common features of this enzyme family (Figure 1(B)).

A Phylogenetic trees for Mrm1 proteins from various species were generated using MEGA5.2 software. Abbreviations for species: Mo (*Magnaporthe oryzae*), NCU (*Neurospora crassa*), FG (*Fusarium graminearum*), BcDW1/BCIN/Bofu (*Botryotinia fuckeliana*), ANIA (*Emericella nidulans*), SNOG (*Phaeosphaeria nodorum*), Sc (*Saccharomyces cerevisiae*), Sp (*Schizosaccharomyces pombe*), Dd (*Dictyostelium discoideum*), HUMAN (*Homo sapiens*), MOUSE (*Mus musculus*). Phylogenetic tree built from full-length Mrm1 protein sequences aligned via MAFFT v7.490 (L-INS-i) and trimmed with TrimAl v1.4.1 (gap threshold = 0.1). Maximum-likelihood tree constructed in MEGA11 using the JTT substitution model with gamma rate variation; bootstrap support values calculated from 1000 independent replicates. B Protein domain predictions for Mrm1 across species were performed using the SMART online tool. Identified domains include SpoU_sub_bind, which is involved in substrate binding, and SpoU_methylase, a domain with methyltransferase activity. C Amino acid sequence similarity among Mrm1 proteins was analyzed using the MAFFT online alignment tool.

A distinctive feature of Mrm1 in filamentous fungi, including the rice blast fungus, is an extended N-terminal sequence beyond the conserved domains, which may play a vital role in its function. This hypothesis prompted further experimental investigation. The high degree of amino acid sequence conservation across species, as shown in Figure 1(C), underscores the importance of Mrm1’s core structure, which is also notably similar in other plant-pathogenic fungi such as *Fusarium graminearum*.

### MRM1 expression patterns correlate with infection stages in M. oryzae development

To gain insights into the potential roles of *MRM1* gene in the rice blast fungus life cycle, including its growth, development, and pathogenicity, we performed expression analysis across various developmental stages. RNA extraction followed by RT-qPCR revealed a dynamic expression pattern for *MRM1*. The gene exhibited relatively low expression during conidium and appressorium formation. However, a significant upregulation occurred from the early infection stage at 18 hours post inoculation (hpi) through to the late infection stage at 48 hpi (Fig. S1). This expression profile implicates a crucial function for *MRM1* in the infectious phase of the fungus.

To elucidate the role of the *MRM1* in the rice blast fungus, we generated a gene knockout via homologous recombination. The split-PCR technique was used to amplify 1.5 kb fragments upstream and downstream of the *MRM1* gene, which were then ligated to the 5’and 3’ ends of the hygromycin resistance gene, *HYG* (Fig. S2A). The PCR products were purified and introduced into the wild-type strain P131 through PEG-mediated transformation, yielding numerous transformants selected by hygromycin resistance. PCR screening of extracted genomic DNA from these transformants verified integration of the 2-kb flanking sequences and absence of the *MRM1* gene. Transformants lacking the *MRM1* band but containing LB2K and RB2K amplicons were confirmed as successfully targeted knockouts. Following this verification process, two knockout strains were obtained and designated as Δ*mrm1-1* and Δ*mrm1-2* (Fig. S2B). We also obtained the complemented transformants, one of which was designated Δ*mrm1/MRM1* for further analysis.

### MRM1 knockout diminishes rice blast fungus growth

To determine the contribution of *MRM1* to the vegetative growth of the rice blast fungus, an essential aspect for its pathogenicity, we inoculated the wild-type (WT), knockout strains Δ*mrm1-1* and Δ*mrm1-2*, and the complemented strain Δ*mrm1/MRM1* onto OTA agar plates (Fig. S3A). Each strain group was tested in triplicate. Following incubation at 28°C in darkness for 120 hours, we measured colony diameters. The knockout strains exhibited reduced growth rates compared to the wild-type and complemented strains. Specifically, the average colony diameters for the wild-type and complemented strains were 4.00 ± 0.08 cm, whereas the knockout strains averaged of 3.72 ± 0.02 cm (Fig. S3B).

Furthermore, we assessed hyphal tip interval – a proxy for growth velocity – by staining with Calcofluor White (CFW) and observing under a fluorescence microscope. The intervals at the hyphal tips of the knockout strains were notably shorter than those of both the wild-type and complemented strains (Fig. S3C and D). Collectively, these findings suggest that *MRM1* promotes vegetative growth in the rice blast fungus.

### MRM1 deficiency enhances conidiation and conidium abnormalities

To determine the impact of *MRM1* on conidiation and conidium morphology, key factors for ecological success of the rice blast fungus, we analyzed the wild-type and the knockout strains Δ*mrm1-1* and Δ*mrm1-2*, along with the complemented strain Δ*mrm1/MRM1*. We examined conidiophore morphology, total conidiation, and the rate of abnormal conidium formation. The knockout strains showed a marked increase in conidiation, averaging 124.84% of the levels in the wild-type and complemented strains (Fig. S4A). This increase did not correlate with significant alterations in conidiophore morphology (Fig. S4B). More notably, the proportion of abnormal conidia – defined as one- and two-celled conidia – was significantly higher in the knockout strains. Whereas the wild-type and complemented strains exhibited abnormal conidial rates below 20%, the knockout strains had approximately 50% abnormal conidia (Fig. S4C and D).

### Knockout of MRM1 leads to reduced virulence in M. oryzae

**Figure 2.**
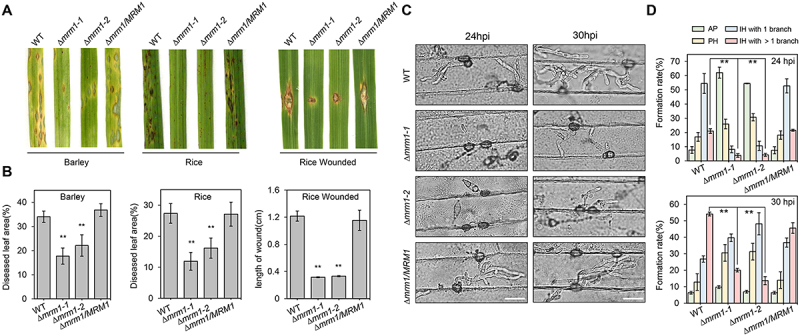
Pathogenicity assessment and infection process of Δ*mrm1* mutants.

To investigate whether *MRM1* is involved in the pathogenic process of the rice blast fungus, we performed barley spray inoculation and rice (LTH) spray and wound inoculation experiments using the WT strain and the Δ*mrm1-1* and Δ*mrm1-2* strains, as well as the complemented strain Δ*mrm1/MRM1*. The knockout strains produced smaller and fewer lesions on barley and rice leaves compared to the wild-type and complemented strains (Figure 2(A,B)), with disease incidence approximately half that of the wild-type strain. Similar results were observed on both hosts, indicating that loss of *MRM1* reduces pathogenicity in the rice blast fungus. Furthermore, wound inoculation on rice showed that knockout strains failed to expand at inoculation sites, whereas the wild-type and complemented strains exhibited spindle-shaped expansion at the wounded sites (Figure 2(A,B)). This suggests that the deletion of *MRM1* impairs the ability of the rice blast fungus to spread within the host cells. Together, these findings demonstrate the importance of *MRM1* for the pathogenicity of the rice blast fungus.

A Inoculation of different strains by barley leaf spray, rice leaf spray, and rice leaf scratch. B Quantitative disease assessment: barley spray inoculation disease area ratio, rice leaf spray inoculation disease area ratio, and rice leaf scratch inoculation lesion expansion length. Statistical significance compared to the wild type is denoted by asterisks (**, *p* < 0.01). C Microscopic examination of the infection process in barley cells at 24 and 30 hpi. AP (appressorium), PH (primary infection hypha), IH (secondary infection hypha). Scale bar, 20 μm. D Statistical histogram depicting the proportion of mycelia at various infection stages at 24 and 30 hpi. Asterisks denote significant differences from the wild type (**, *p* < 0.01).

### MRM1 deletion impedes infectious hyphal expansion in host cells

The progression from appressoria to infectious hyphae is a critical phase in the invasion of host cells by the rice blast fungus, facilitating nutrient acquisition and reproduction. To determine the impact of *MRM1* knockout on this process, we examined the infectious hyphal expansion on barley epidermal cells. At 24 hpi, over 50% of the infectious hyphae from the wild-type and complemented strains had branched at least once, with approximately 20% exhibiting multiple branches. In contrast, nearly 60% of knockout strain’s structures remained at the appressorium stage, and only about 20% had initiated branching (Figure 2(C,D)). By 30 hpi, the wild-type and complemented strains showed advanced multi-branching, whereas less than half of knockout hyphae had a single branch, and multi-branching hyphae were below 20% (Figure 2(C,D)). These observations suggest that *MRM1* is essential for timely infectious hyphal expansion within host cells. The delayed hyphal branching in the knockout strains correlates with the significant loss of pathogenicity, providing a plausible explanation for the reduced ability to invade host cells in the absence of *MRM1*.

### MRM1 knockout delays appressorium formation

**Figure 3.**
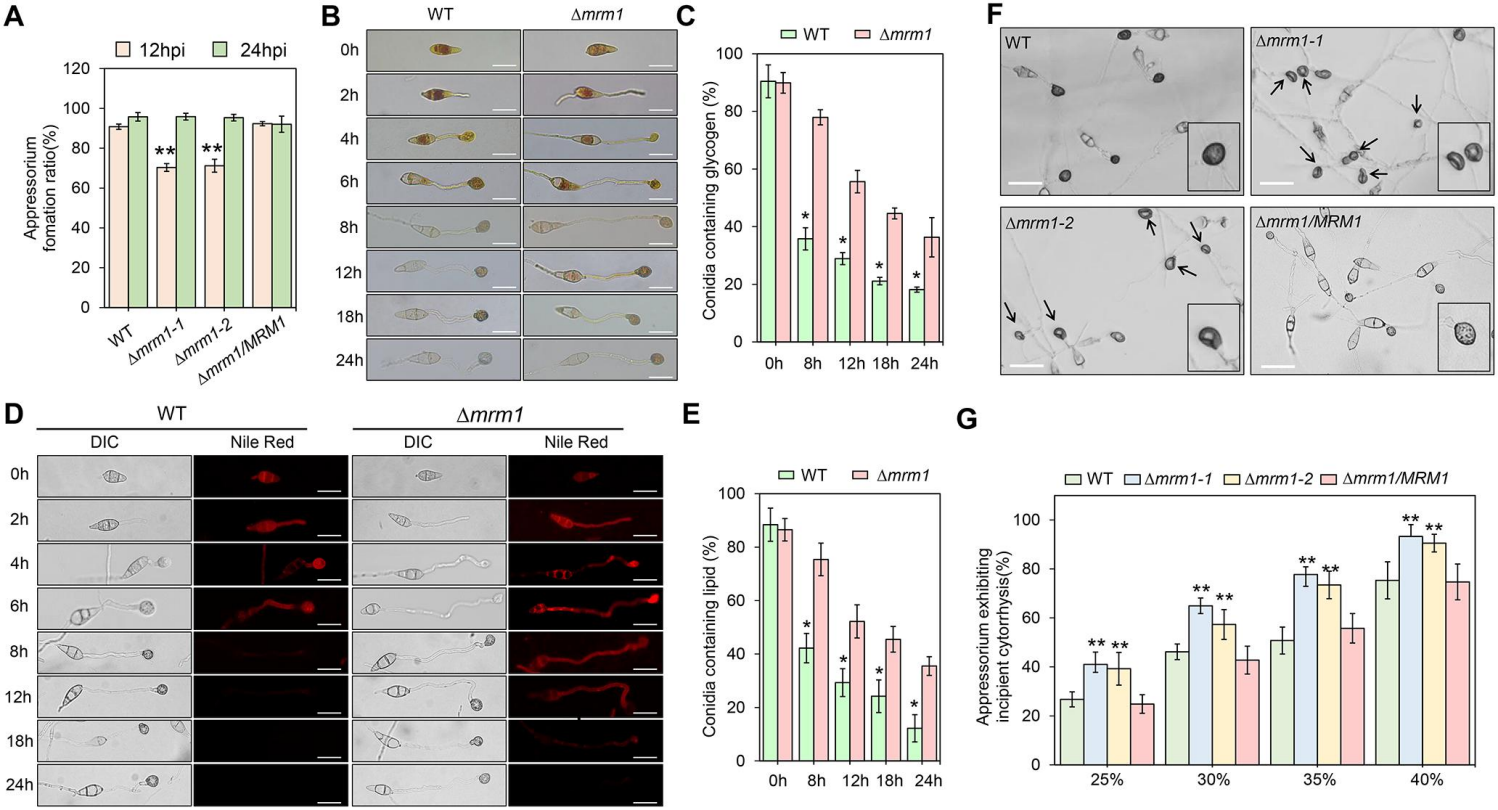
Analysis of appressorium formation in Δ*mrm1* mutants.

The role of *MRM1* in the pathogenicity prompted an investigation into its potential influence on appressorium formation, a critical step in the infection process. We assessed appressorium formation efficiency in the wild-type strain and the knockout strains Δ*mrm1-1* and Δ*mrm1-2*, along with the complemented strain Δ*mrm1/MRM1* on hydrophobic glass slides. The knockout strains showed an initial lag, with a formation rate approximately 30% lower than the wild-type at 12 hpi. However, by 24 hpi, the knockout strains had caught up, achieving rates comparable to the wild-type (Figure 3(A)), with nearly all conidia having developed into appressoria. This suggests that although *MRM1* deficiency slows the initial appressorium development, it does not impede the overall ability of the fungus to form appressoria.

A Bar graph depicting the rate of attachment cell formation at 12 and 24 hours post inoculation (hpi). Statistical significance compared to the wild type is denoted by asterisks (**, *p* < 0.01). Scale bar, 20 μm. B Microscopic examination of glycogen metabolism during appressorium formation and maturation. Scale bar represents 20 μm. C Quantitative analysis of conidia and appressorium stained in the context of glycogen metabolism. Asterisks indicate significant differences from the wild type (*, *p* < 0.05).DObservation of lipid metabolism during the development of appressorium. Scale bar represents 20 μm. E Proportion of conidia and appressorium stained in lipid metabolism processes, with significant differences marked by asterisks (*, *p* < 0.05). F Microscopic observation of appressorium collapse under various concentrations of PEG8000 stress, with an arrow indicating a collapsed appressorium. Scale bar, 20 μm. G Bar chart showing the appressorium collapse rate under various concentrations of PEG8000, with asterisks highlighting significant differences compared to the wild type (**, *p* < 0.01).

### MRM1 knockout impairs lipid and glycogen metabolism

Given the observed delay in appressorium formation in the *MRM1* knockout strain, we investigated potential disruptions in maturation of appressoria. Metabolic analyses focused on lipid and glycogen metabolism within appressoria of the wild-type and Δ*mrm1* strains. We used Nile red staining to visualize lipid body distribution and I_2_/KI staining to assess glycogen presence as an indicator of metabolic activity.

I_2_/KI staining revealed a clear difference in glycogen metabolism: glycogen in the WT strain was nearly exhausted by 8 hpi, whereas it persisted beyond 12 hpi in the knockout strain (Figure 3(B,C)). This suggests a defect in glycogen metabolism due to the absence of *MRM1*. Consistent with this finding, Nile red staining showed a similar pattern in lipid metabolism. The wild-type strain displayed a significant reduction in lipid bodies by 8 hpi, whereas the knockout strain retained substantial lipid even at this stage (Figure 3(D,E)), indicating impaired lipid metabolism. These metabolic disruptions suggest that *MRM1* knockout results in dysfunctional appressoria, potentially impeding their ability to invade host cells and contributing to the observed reduction in pathogenicity.

### MRM1 deletion results in reduced turgor pressure in appressoria

Prior research indicated impaired utilization of glycogen and lipids during appressorium formation in the *MRM1* knockout strain. Given that glycerol production from these sources is essential for generating the turgor pressure required for host penetration, we examined osmotic pressure of appressoria in the WT, knockout strains Δ*mrm1-1* and Δ*mrm1-2*, and the complemented strain Δ*mrm1/MRM1* using varying concentrations of PEG8000. The knockout strains showed a higher incidence of collapsed appressoria in under all PEG8000 concentrations compared to the wild-type and complemented strains (Figure 3(F,G)), indicating significantly lower osmotic pressure within their appressoria. These results underscore the importance of Mrm1 in the regulation of turgor pressure in rice blast fungus appressoria. The compromised functionality of appressoria in the knockout strains likely contributes to their diminished capacity to infect host tissues, consistent with the observed attenuation in pathogenicity.

### Heterologous complementation of M. oryzae MRM1 knockout with N. crassa MRM1

**Figure 4.**
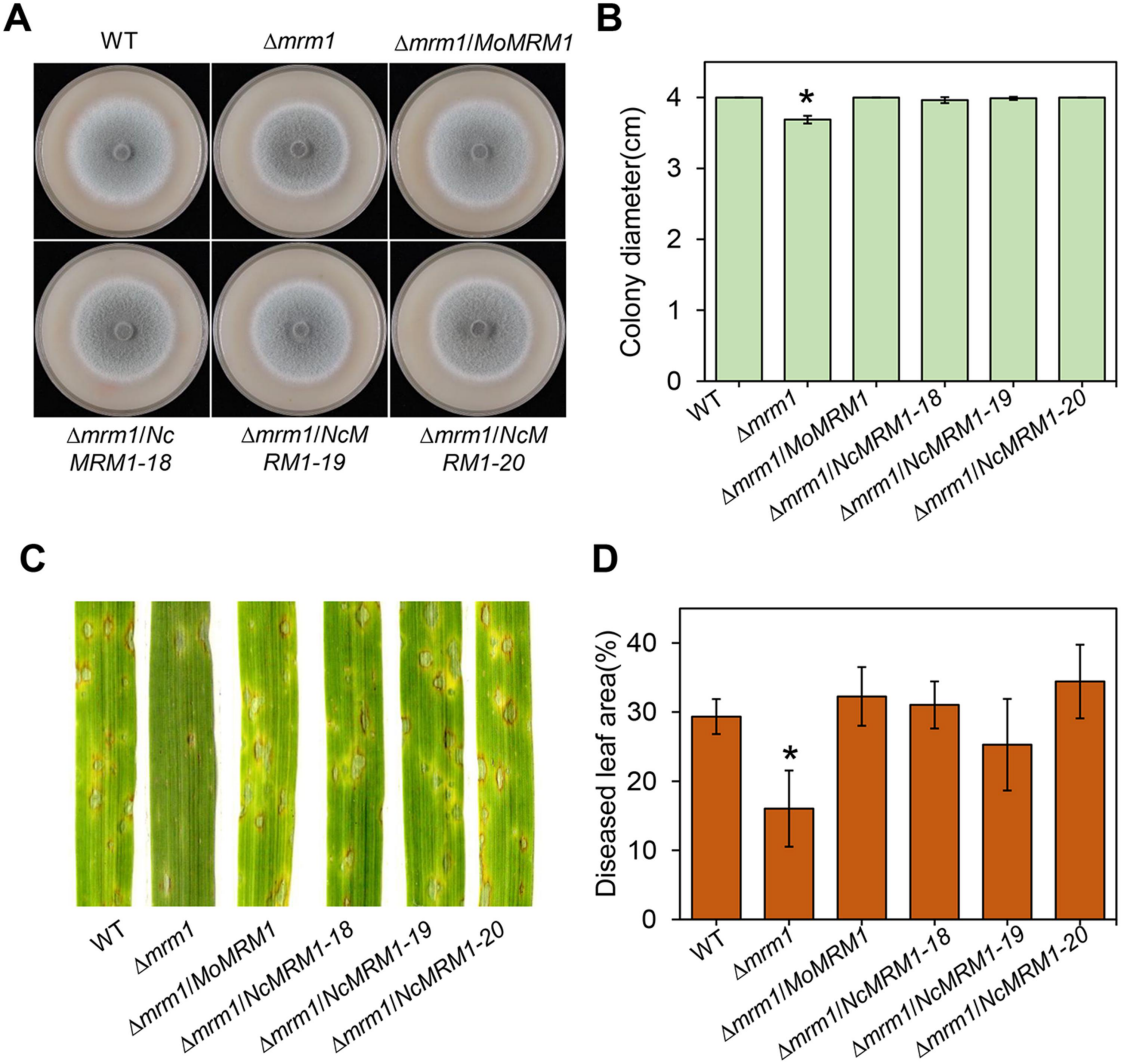
Complementation of the *M. oryzae* ∆*mrm1* mutant with *N. crassa MRM1*.

Collaborative studies identified Mrm1 in *N. crassa* as an rRNA methyltransferase that catalyzes 2’-O-methylation of guanine at a specific position in the 23S rRNA of the mitochondrial large ribosomal subunit [15]. The Mrm1 proteins of the rice blast fungus and *N. crassa* share high sequence homology (Figure 1(A,B)). To further explore Mrm1 function in *M. oryzae*, we constructed a complementation vector containing the *N. crassa MRM1* gene and introduced it into the *M. oryzae MRM1* knockout strain. We selected positive transformants through fluorescence screening, yielding three strains: Δ*mrm1/NcMRM1-18*, Δ*mrm1/NcMRM1-19*, and Δ*mrm1/NcMRM1-20*. We then assessed growth rate and pathogenicity in these strains. The complementation analysis demonstrated that Mrm1 from *N. crassa* Mrm1 fully rescues the growth and pathogenicity defects of the *M. oryzae MRM1* knockout (Figure 4(A-D)), indicating functional conservation of Mrm1 between these two fungi and highlighting the importance of this putative rRNA methyltransferase in fungal biology.

A growth assessment of the *NcMRM1* complemented *M. oryzae*strains. Observations were made on OTA plates for various strains after 120 hours post inoculation (hpi). B Bar chart displaying the statistical analysis of growth diameters, with asterisks marking significant differences (*, *p* < 0.05). C Pathogenicity evaluation of the *NcMRM1* complemented *M. oryzae* strains. Schematic representation of disease lesion formation following spray inoculation on barley leaves with various strains. D Histogram illustrating the ratio of diseased area to total leaf area, with asterisks indicating significant differences (*, *p* < 0.05).

### Domain truncation in Mrm1 protein highlights N-terminal importance for fungal pathogenicity

**Figure 5.**
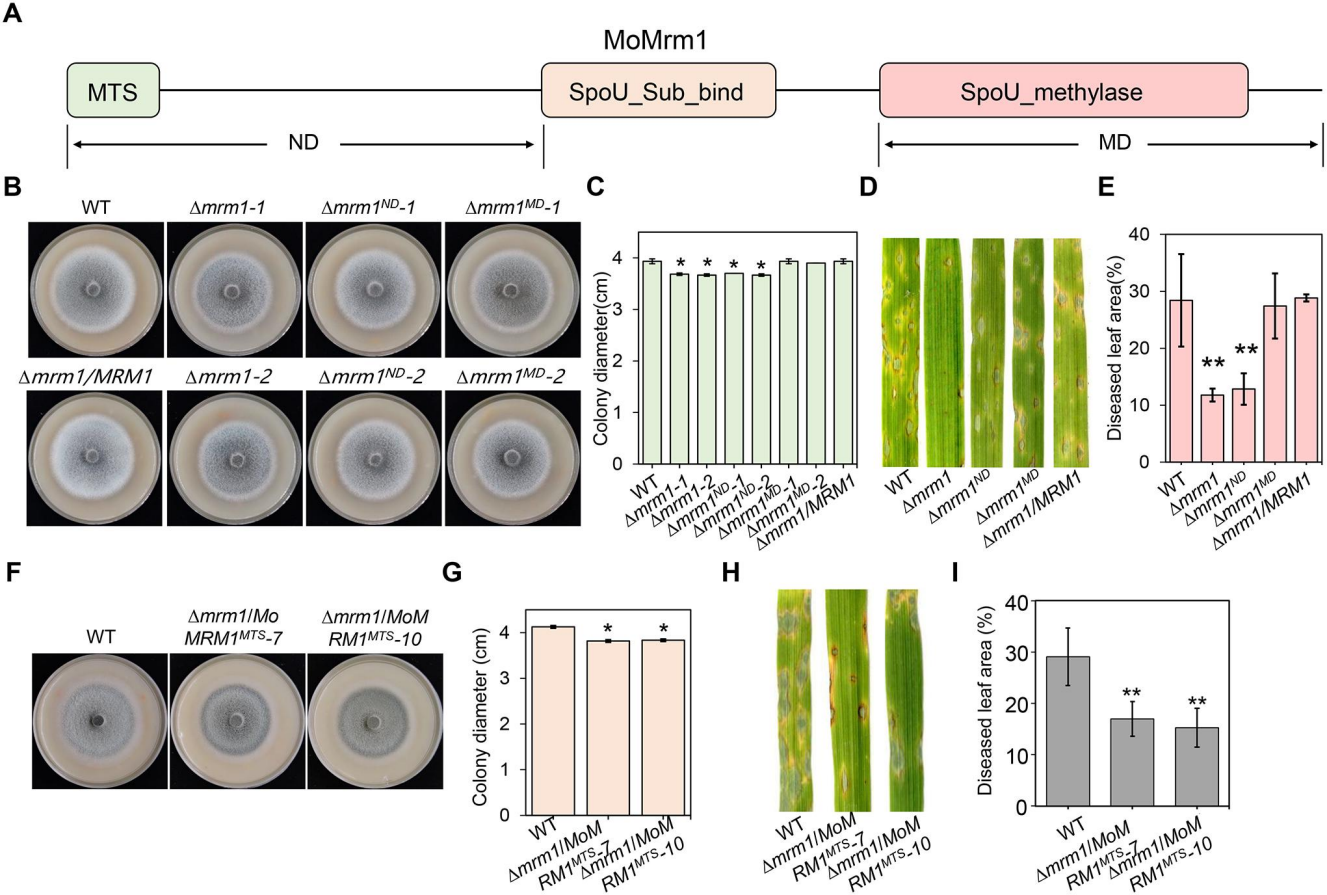
Growth and pathogenicity of Mrm1 protein truncated strains.

Protein domain structure predictions identified a lengthy N-terminal sequence in Mrm1 of filamentous fungi, including the rice blast fungus, hypothesized to be functionally significant. To delineate the role of various domains, we created a series of truncation mutants: ND (lacking the N-terminal sequence) and MD (lacking the C-terminal methyltransferase domain). We integrated these mutants into GFP vectors under the control of their native promoters (Figure 5(A)) and selected transformants based on fluorescence, designating them Δ*mrm1*^*ND*^ and Δ*mrm1*^*MD*^.

A schematic representation of the predicted Mrm1 protein domains, with ND for the N-terminal sequence, MTS for the mitochondrial targeting sequence, MD for the C-terminal sequence containing the methyltransferase domain, and the active site R highlighted in red. B Colony diameter measurements of the mutant strains on OTA plates at 120 hpi. C Histogram of the colony diameter statistics, with asterisks marking significant differences (*, *p* < 0.05). D Colony diameter comparison between the wild-type and the ∆*mrm1*^*MTS*^strain at 120 hpi on OTA plates. E Histogram of the colony diameter statistics for the wild-type and ∆*mrm1*^*MTS*^strain, with asterisks indicating significant differences (**, *p* < 0.01). F Barley leaf spray inoculation with the series of mutant strains. G Bar chart of the barley spray inoculation incidence area proportion, with asterisks indicating significant differences (*, *p* < 0.05). H Barley leaf spray inoculation with the wild-type and ∆*mrm1*^*MTS*^strains. I Bar chart of the incidence area proportion for barley leaves spray-inoculated with the wild-type and ∆*mrm1*^*MTS*^strains, with asterisks indicating significant differences (**, *p* < 0.01).

Growth rate analysis showed that the Δ*mrm1*^*ND*^ strain phenocopied the knockout growth deficiency, whereas Δ*mrm1*^*MD*^ strains grew similarly to the wild-type and complemented strains (Figure 5(B,C)). The preservation of normal growth in the MD strains was surprising, given the expected impact of losing catalytic domain. Notably, the N-terminal truncation strain Δ*mrm1*^*ND*^ displayed a growth phenotype identical to the knockout, underscoring the importance of the N-terminus for Mrm1 functionality.

Further assessment of these mutants via spray inoculation on barley confirmed the growth findings, with the Δ*mrm1*^*ND*^ strain showed severe loss of pathogenicity, similar to the knockout strain, whereas the Δ*mrm1*^*MD*^ strains retained pathogenicity comparable to the wild-type and complemented strains (Figure 5(D,E)). These results reinforce that the N-terminal sequence is pivotal for the pathogenicity of the rice blast fungus.

### MTS removal in Mrm1 disrupts mitochondrial localization and fungal pathogenicity

Further investigation into the N-terminal sequence of Mrm1, which is implicated in mitochondrial localization, expanded our understanding of its role in the rice blast fungus. Mitochondrial proteins, synthesized by the cytoplasmic system, possess a mitochondrial targeting sequence (MTS) that facilitates their import into mitochondria. Given co-localization findings suggesting mitochondrial residence of Mrm1, we predicted the MTS of Mrm1 using MitoFates. We then constructed a GFP vector excluding the MTS and transformed it into the knockout strain, yielding transformants designated Δ*mrm1*^*MTS*^, identified through fluorescence screening.

Growth rate and pathogenicity assessment showed that the Δ*mrm1*^*MTS*^ strain phenocopied the knockout and Δ*mrm1*^*ND*^ strains, with slowed growth and attenuated pathogenicity (Figure 5(F–I)). These findings suggest that absence of the MTS impedes normal mitochondrial translocation of Mrm1, thereby affecting pathogenicity. Collectively, the N-terminus of Mrm1 appears multifunctional, extending beyond the role of the MTS in dictating subcellular localization. The minimal impact of the methyltransferase catalytic domain on Mrm1 function hints at additional crucial elements within the N-terminal sequence that are vital for the protein’s role in pathogenicity of the rice blast fungus.

### MTS deletion in Mrm1 mutants disrupts localization and impairs function

**Figure 6.**
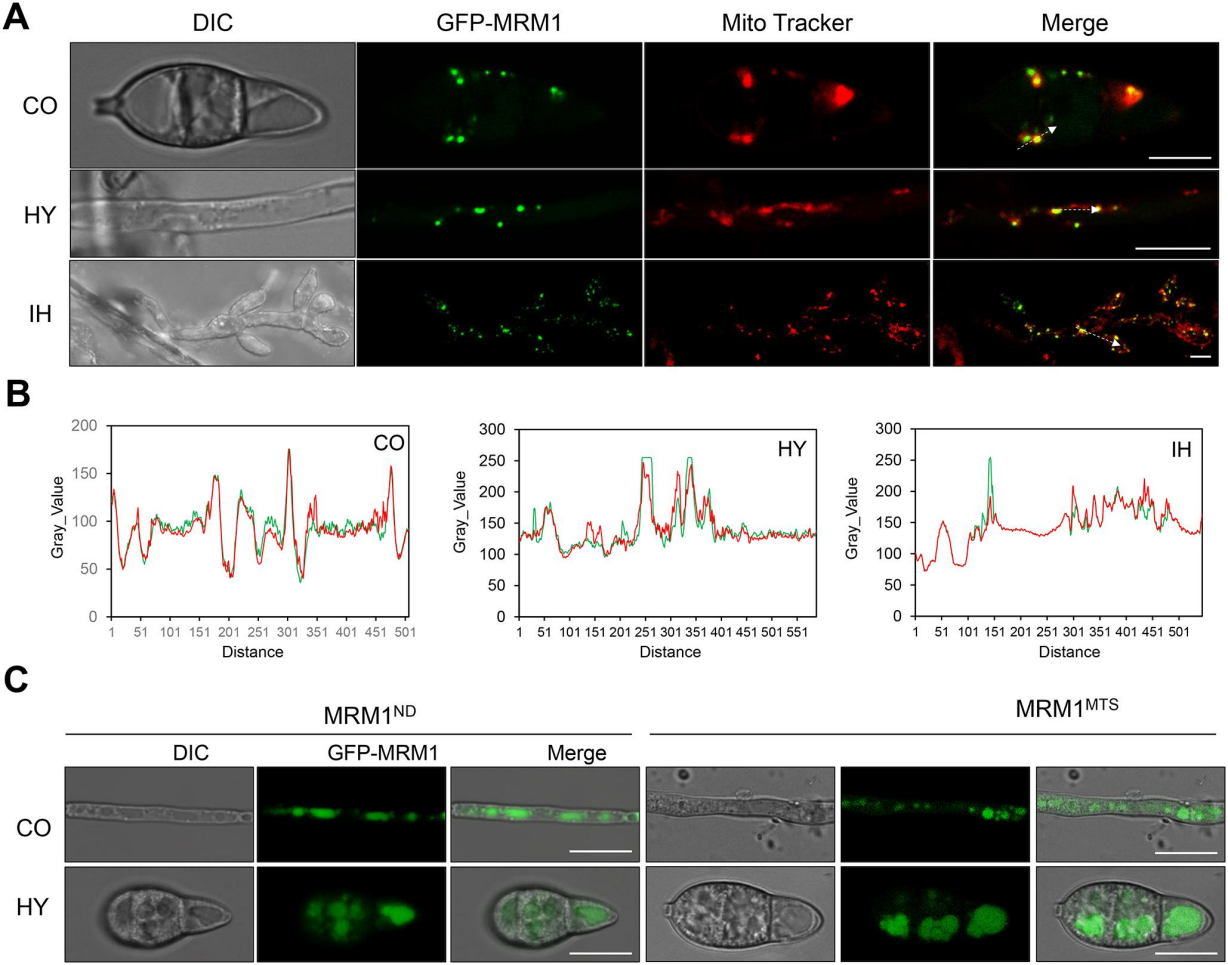
Subcellular localization of the Mrm1 protein.

To elucidate the cellular role of Mrm1 in the rice blast fungus, we introduced a GFP-Mrm1 construct into the wild-type strain. We identified positive transformants and examined their fluorescence by confocal microscopy, which revealed a punctate pattern suggestive of mitochondrial localization. We confirmed this by co-staining conidia (CO), vegetative hyphae (HY), and infectious hyphae (IH) with Mito Tracker, a mitochondria-specific dye. Co-localization analysis showed that the WT/GFP-Mrm1 fluorescence overlapped significantly with the Mito Tracker signal, indicating mitochondrial localization with minor cytoplasmic distribution (Figure 6(A,B)). This suggests a primary mitochondrial role for Mrm1, with potential additional functions in the cytoplasm.

A Co-localization of IH samples with Mito Tracker visualized under the microscope. Conidia (CO); Mycelial hyphae (HY); Infection hypha (IH). Scale bar, 10 µm. B Fluorescence intensity profiles of the co-localized samples from three independent experiments were plotted using ImageJ. C Observation of subcellular localization for the ∆*mrm1*^*ND*^and ∆*mrm1*^*MTS*^strains. Conidia (CO); Mycelial hyphae (HY); Infection hypha (IH). Scale bar represents 10 µm.

The similar phenotypes of Δ*mrm1*^*ND*^ and Δ*mrm1*^*MTS*^ strains in preliminary tests motivated further exploration of MTS function in directing proteins to mitochondria. We used confocal microscopy to assess the subcellular localization in hyphae and conidia of these strains. The results supported the hypothesis that MTS absence leads to mislocalization: instead of mitochondrial localization, GFP fluorescence abnormally accumulated in vacuolar compartments of both mutant strains (Figure 6(C)). This mislocalization likely results from deletion of the MTS, which prevents normal mitochondrial import and causes cytoplasmic accumulation. GFP signal from MTS-truncated Mrm1 accumulates prominently within vacuolar compartments, consistent with mistargeting and potential vacuolar turnover of mislocalized mitochondrial protein. Future pharmacological assays with vacuolar protease and autophagy inhibitors will confirm degradation biochemically.

### Mrm1 regulates mitochondrial morphology affecting fungal virulence

**Figure 7.**
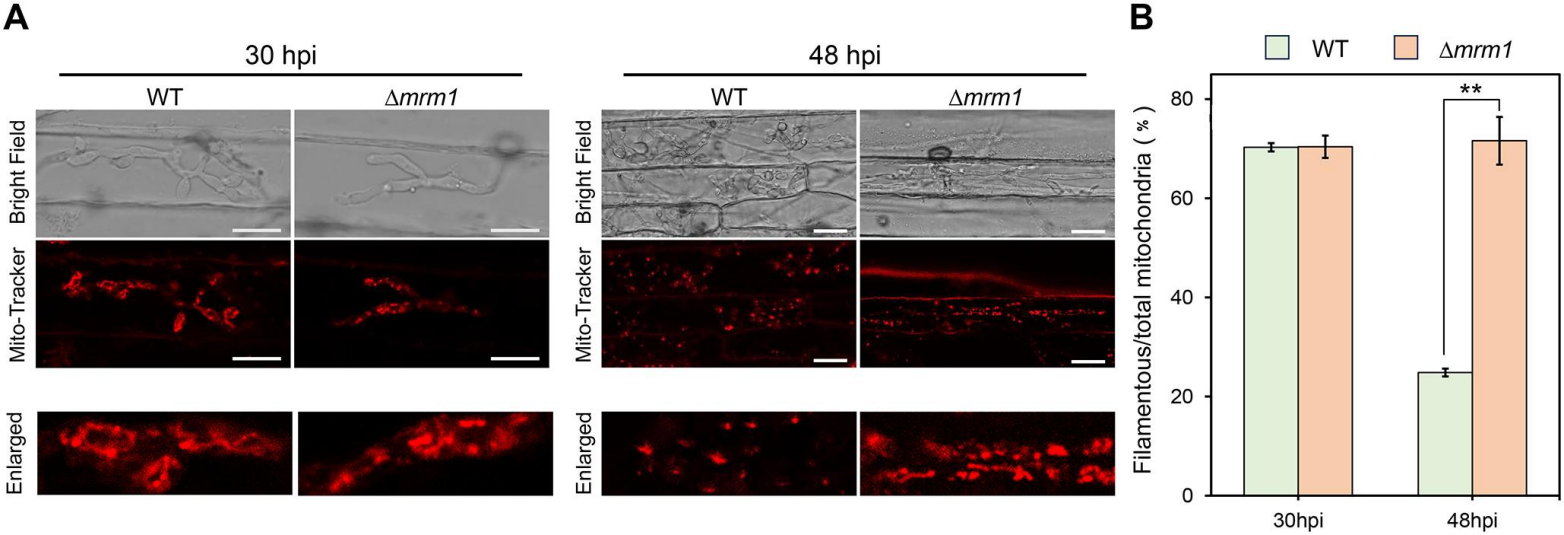
Mrm1 is involved in the formation of mitochondrial morphology of *M. oryzae.*

Given the established mitochondrial localization of Mrm1, we hypothesized a role in mitochondrial function. Mitochondrial dynamics, involving fission and fusion, are crucial for maintaining normal morphology, which is essential for virulence of the rice blast fungus. Absence of proteins pivotal to these processes, such as Dnm1 and Fzo1, causes mitochondrial abnormalities and diminish fungal pathogenicity. To investigate the impact of *MRM1* deletion on mitochondrial dynamics, we stained infectious hyphae with the mitochondrial-specific dye MitoTracker and examined mitochondrial morphology during infection by confocal microscopy. At 30 hpi, both wild-type and knockout strains exhibited a mix of filamentous and punctate mitochondrial patterns. However, by 48 hpi, a distinction emerged: while wild-type mitochondria predominantly displayed a punctate morphology, knockout mitochondria were primarily filamentous, indicative of mitochondrial disarray (Figure 7(A,B)). These findings implicate Mrm1 in the regulation of mitochondrial fission and fusion. The observed mitochondrial dysfunction in the knockout strain likely contributes to the attenuated virulence of the rice blast fungus.

A Observation of mitochondrial morphology in both mutant and wild-type strains at various time points. Infection hypha samples at 30 hpi and 48 hpi with Mito Tracker visualized under the microscope. Scale bar, 20 µm. B Statistical analysis showing the proportion of linear mitochondria among the total mycelia infected by the wild-type and mutant strains, with asterisks indicating significant differences (**, *p* < 0.01).

### MRM1 deletion impairs mitochondrial function and reduces global translation efficiency

**Figure 8.**
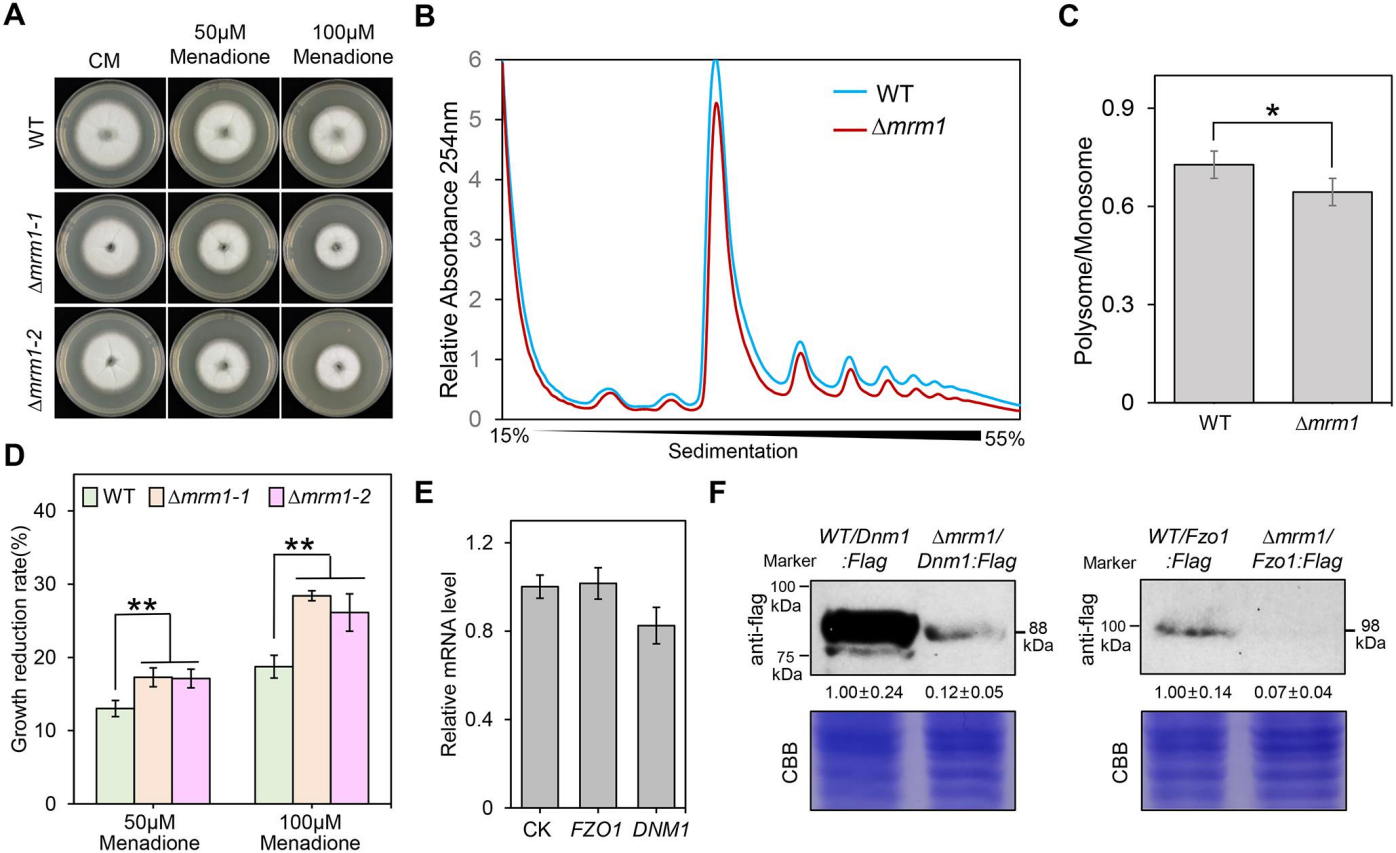
Mrm1 regulates the translation efficiency of mitochondrial protein Fzo1 and Dnm1.

The mitochondrial localization of Mrm1 suggests its involvement in the translation of proteins encoded by mitochondrial DNA, which are crucial for the electron transport chain. To test whether *MRM1* deletion causes mitochondrial dysfunction, we compared the oxidative stress tolerance of wild-type and Δ*mrm1* strains using menadione, a redox-cycling agent that induces broad mitochondrial reactive oxygen stress. The knockout strains showed increased inhibition rates at 50 µM and 100 µM menadione, indicating greater sensitivity than the wild-type (Figure 8(A,B)). This suggests that Mrm1 contributes to assembly or function of the mitochondrial electron transport chain, as its absence results in heightened menadione sensitivity.

A Evaluation of the sensitivity to Menadione in both wild-type and mutant strains. Growth conditions were examined on CM plates after 120 hours post inoculation (hpi) with two Menadione concentrations, 50 µM and 100 µM. B Translational efficiency was assessed using Polysome Profiling with a sucrose gradient ranging from 15% to 55%. All polysome/monosome peak area ratios were normalized to matched input RNA absorbance values, and RNA integrity was confirmed by agarose electrophoresis for all tested samples to exclude loading and degradation interference. The 40S peak indicates small ribosomal subunits, the 60S peak represents large ribosomal subunits, the 80S peak denotes single ribosomes, and the subsequent polysome peaks correspond to multiple ribosomes engaged in translation. C Measurement of the colony growth diameters to determine the inhibition rates at varying concentrations. An asterisk denotes a statistically significant difference (*, *p* < 0.05). D The histogram presents the ratio of the area under the curve for polysomes to that for single ribosomes, with significant differences marked by an asterisk (**, *p* < 0.01). E The expression levels of the DNM1 and FZO1 genes in mutant strains were quantitatively compared to those in the wild-type strain. F Western blot analysis was conducted to determine the relative protein levels of Dnm1 and Fzo1 between the wild-type and mutant strains, demonstrating that Mrm1 affects the steady-state protein levels of Dnm1 and Fzo1, likely through its role in translation.

Given Mrm1’s role as a putative rRNA methyltransferase in modulating mRNA translation, we investigated the impact of *MRM1* deletion on the global translation in the rice blast fungus using polysome profiling. This method compares translation rates between strains by calculating the ratio of ribosome-occupied areas in polysomes to those of single ribosomes. All polysome-to-monosome ratios were normalized to input RNA absorbance to avoid loading artifacts, and agarose gel checks confirmed equivalent RNA integrity across samples. The results revealed slightly reduced overall translation in the knockout strain compared to the wild-type (Figure 8(C,D)), indicating that Mrm1 is important for maintaining normal translational activity.

### MRM1 deletion decreases translation of mitochondrial fission-fusion proteins

Given Mrm1’s role as a putative rRNA methyltransferase and its potential influence on ribosome-related processes, we focused on the impact of *MRM1* deletion on the protein levels of the key mitochondrial fission-fusion regulators Dnm1 and Fzo1 in the rice blast fungus. Protein abundance is governed by both transcriptional and translational regulation, with alteration at either level potentially affecting protein quantity. An initial comparison of *DNM1* and *FZO1* gene expression between wild-type and knockout strains revealed no significant differences (Figure 8(E)), directing the investigation toward protein translation.

We constructed expression vectors 3×Flag-Dnm1 and 3×Flag-Fzo1 and transformed them into both wild-type and Δ*mrm1* strains. Western blot analysis showed that, under equal loading, Dnm1 and Fzo1 protein levels were markedly reduced in the knockout strain compared to the wild-type (Figure 8(F)). This indicates that Mrm1 affects the steady-state protein levels of Dnm1 and Fzo1, likely through its role in translation, without altering transcriptional abundance of *DNM1* and *FZO1*. Consequently, abnormal mitochondrial morphology in infectious hyphae of the knockout strain and a corresponding decrease in virulence likely result from reduced Dnm1 and Fzo1 translation.

## Discussion

This work characterizes the mitochondrial putative rRNA methyltransferase Mrm1 and demonstrates its essential roles in vegetative growth, conidiation, and virulence of *M. oryzae*. Mrm1 is vital for fungal growth, conidium production, and pathogenicity. The evolutionary conservation of the protein and its unique N-terminal sequence highlight its significant role in fungal biology. Additionally, Mrm1 is indispensable for timely appressorium formation, maintenance of turgor pressure required for host penetration, and infectious hyphal expansion. More intriguingly, Mrm1 is crucial for mitochondrial morphology and function, which directly relates to fungal virulence. It localizes specifically to mitochondria, where it regulates mitochondrial dynamics, particularly the equilibrium between the processes of fission and fusion. These processes are essential for preserving the normal form and function. Our results suggest that rRNA methylation by Mrm1 affects translation of proteins vital for mitochondrial function. This translational defect disrupts mitochondrial energy homeostasis and impairs fungal infection capacity. The findings highlight the potential of Mrm1 as a target for managing rice blast disease and contribute to a more comprehensive understanding of fungal pathogenesis mechanisms.

Over the past decade, RNA modifications have attracted extensive research interest and have seen a consistent increase in scientific interest [16]. m^6^A and m^5^C have emerged as the most widely studied modifications, with research focused on mRNA [17]. Technological advancements suggest that modifications in low-abundance RNAs will become more apparent in the coming years [16]. In contrast, rRNA modifications, such as 2’-O-methylation (Nm) and pseudouridine (Ψ) remain less explored [18]. The enzymes responsible for these modifications fall into two categories: those guided by box C/D snoRNPs and box H/ACA snoRNPs, and those directed by individual enzymes [19]. The rRNA methyltransferase Mrm1 belongs to the latter group. Studies have demonstrated its role in catalyzing Gm1145 modification on the human ribosomal large subunit [20], Gm2270 on the yeast ribosomal large subunit RNA [21], and Gm2251 on the *Escherichia coli* 23S rRNA [22]. Previous studies on Mrm1 across species have concentrated on the types of modifications it catalyzes rather than its biological functions. To date, the biological role of this modification enzyme in the rice blast fungus, a plant pathogen, has remained unexplored.

In this study, we identified the Mrm1 protein in the rice blast fungus through homologous protein sequence alignment with its human and yeast counterparts. Using a reverse genetics strategy, we generated two knockout strains exhibiting consistent phenotypes via homologous recombination. Although direct sequencing has not been conducted in the rice blast fungus, our collaborators performed Nm-seq analysis in *N. crassa* and established that NcMrm1 catalyzes Gm2453 on 23S rRNA. To confirm that Mrm1 functions as an Nm modification enzyme in the rice blast fungus, we introduced *NcMRM1* from *N. crassa* into the Δ*mrm1* strain through heterologous complementation. Restoration of the phenotype in the complemented strain indicates that Mrm1 in the rice blast fungus possesses Nm modification activity.

The putative rRNA methyltransferase enzyme Mrm1 significantly impacts mitochondrial structure and function in *M. oryzae*. Mrm1 localizes to mitochondria and regulates mitochondrial dynamics, specifically the balance between fission and fusion. These processes are crucial for maintaining normal mitochondrial morphology and function. Upon *MRM1* inactivation, the fungus exhibits irregular mitochondrial morphology, with increased filamentous (fused) mitochondria particularly during later infection stages. This indicates that Mrm1 is essential for proper mitochondrial segmentation and distribution. Notably, mitochondrial fission-fusion status relates to pathogenicity [5]. Additionally, MRM1 deletion reduces synthesis of key proteins involved in mitochondrial fission and fusion, including Dnm1 and Fzo1. Decreased levels of these proteins disrupt normal mitochondrial dynamics, potentially reducing energy production and buffering capacity. Polysome profiling further decreased global translation in the Δ*mrm1* strain, consistent with reduced Dnm1 and Fzo1 protein abundance. These capabilities are critical for host cell invasion and infection initiation.

These correlative results indicate Mrm1 affects steady-state protein levels of Dnm1 and Fzo1, likely via its function in translational homeostasis, though direct evidence linking rRNA methylation to selective translation of *DNM1*/*FZO1* transcripts is currently lacking. We rely on menadione growth inhibition to reflect altered mitochondrial stress response in Δ*mrm1* mutants rather than drawing definitive conclusions about impaired electron transport chain activity. Follow-up experiments, including rotenone treatment, mitochondrial membrane potential staining, and cellular ATP quantification, will further characterize mitochondrial respiratory dysfunction in the *MRM1*-deleted strain. This, in turn, could indirectly impact overall energy metabolism and the pathogenicity of the rice blast fungus. The study highlights the complex interplay among rRNA modification, mitochondrial homeostasis, and the virulence of fungi.

In the rice blast fungus, the Mrm1 protein contains a notably longer N-terminal segment than human and mouse homologs. We generated Δ*mrm1*^*ND*^ (N-terminal deletion) and Δ*mrm1*^*MD*^ (catalytic methyltransferase domain deletion) truncation mutants. Phenotypic assays showed Δ*mrm1*^*ND*^ recapitulated all mutant defects, whereas Δ*mrm1*^*MD*^ behaved like the wild-type. The normal growth and virulence of Δ*mrm1*^*MD*^ challenge the assumption that methyltransferase activity alone drives Mrm1 function, pointing to N-terminal autonomous roles such as mitochondrial structural scaffolding or protein interactions. We propose two models: (i) the N-terminus alone sustains mitochondrial ribosome assembly independent of the catalytic domain; (ii) N-terminal motifs mediate critical mitochondrial protein interactions without requiring methylation. Thus Mrm1’s effect on Dnm1 and Fzo1 protein levels may involve both methyltransferase-dependent and -independent mechanisms, with the N-terminal region playing a previously unrecognized essential role. Follow-up active-site point mutants will verify whether methyltransferase activity is indispensable for mitochondrial dynamics and virulence. This suggests that the N-terminal sequence is crucial for Mrm1 function, contrary to the catalytic domain’s importance. We next investigated the N-terminal role in subcellular localization and identified the MTS as necessary for Mrm1’s mitochondrial function. A strain lacking the MTS, Δ*mrm1*^*MTS*^, exhibited a phenotype akin to Δ*mrm1*^*ND*^, confirming that mitochondrial entry is pivotal for Mrm1 function. Our current confocal imaging provides only correlative evidence for vacuolar enrichment of MTS-deleted Mrm1; we lack direct biochemical proof to confirm that the vacuolar GFP signal originates from protein degradation mediated by vacuolar proteases or autophagy. Future pharmacological treatments with vacuolar protease and autophagy inhibitors will verify this tentative model and distinguish mistargeting from protein turnover. The functional motifs within the N-terminus outside the MTS remain uncharacterized; future mutagenesis will pinpoint key residues governing Mrm1 activity. This approach will clarify the contribution of each domain to Mrm1’s biological role in the rice blast fungus.

## Conclusion

This study pioneers investigation of the single-enzyme putative rRNA methyltransferase Mrm1, establishing its critical role in the growth and pathogenicity of the rice blast fungus. We also demonstrate that Mrm1 indirectly affects mitochondrial fission-fusion by modulating protein levels of Dnm1 and Fzo1, highlighting its significance in mitochondrial biology. This work contributes to a clearer understanding of pathogenic mechanisms in the rice blast fungus, and may inform future prevention and control strategies.

## Materials and methods

### Fungal strains and growth conditions

The wild-type strain P131 of *M. oryzae* served as the reference strain in this study [23]. All strains, detailed in Supplementary Table S1, were grown on oatmeal tomato agar (OTA) plates at 28°C. For RNA and protein extraction, cultures were maintained in liquid CM medium for 36 hours at 28°C. Colony growth and conidiation were assessed using previously established methods. Conidia from 7-day-old colonies grown on OTA plates were collected in 0.025% Tween-20 for inoculation. Sensitivity assays were performed by adding compounds to CM plates at various concentrations, followed by incubation at 28°C. Growth inhibition was evaluated by measuring colony diameters at 5 days post inoculation (dpi) and calculating inhibition rates.

### Gene knockout and complementation

We generated a targeted *MRM1* knockout in *M. oryzae* via homologous recombination. The selectable marker gene hygromycin phosphotransferase (HYG) replaced the *MRM1* gene. Primers were designed to amplify approximately 2-kb regions both upstream and downstream of the *MRM1* (Supplementary Table S2). These genomic fragments were cloned and fused with the *HYG* gene to generate the knockout construct. The cassette was introduced into the wild-type strain via polyethylene glycol (PEG)-mediated protoplast transformation. Transformants were selected on hygromycin-containing medium (Roche Diagnostics, Indianapolis, IN, USA), and successful *MRM1* replacement was confirmed by PCR with gene-specific primers.

For complementation, we amplified a 2-kb promoter region, the full *MRM1* coding sequence, and a ~ 500-bp downstream segment from the P131 genome. This fragment was cloned into the pGTN vector (Supplementary Table S3) and transformed into the Δ*mrm1* strain. Complemented transformants (Δ*mrm1*/*MRM1*) were selected on neomycin at 400 μg/mL.

### Growth rate determination and hyphal tip observation

We monitored in vitro growth rate of the wild-type and Δ*mrm1* mutant strains by inoculating them OTA and incubating at 28°C. Hyphal tip morphology and septum formation were examined using Calcofluor White (CFW, Sigma-Aldrich, St. Louis, MO, USA), a fluorescent dye that binds to cellulose and chitin, to highlighting the hyphal structure under a fluorescence microscope.

### Conidiation capacity assay

We induced and assessed conidiation cultivating the strains on oatmeal tomato agar (OTA) plates, which promote conidiophore development and conidium release. Conidia were washed with sterile water to obtain a single-cell suspension, and conidium number was determined using a hemocytometer.

### Appressorium formation and turgor pressure measurement

We monitored appressorium formation on hydrophobic surfaces and evaluated formation rates microscopically at various time points after inoculation. To measure the turgor pressure within appressoria, we performed a cytorrhysis assay. Conidia at 5 × 10^5^/ml were placed onto hydrophobic coverslips and incubated in darkness at 28°C under high humidity. After 24 hours, appressoria were treated with polyethylene glycol (PEG) 8000 solutions at 25%, 30%, 35%, and 40% (w/v) for 5 minutes. The percentage of collapsed appressoria was determined microscopically, with at least 100 conidia assessed per concentration.

### Pathogenicity assays and infection process observation

Seeds of rice (*Oryza sativa* L. ssp. *japonica* cv. Lijiangxintuanheigu, LTH) were obtained from the Institute of Crop Sciences, Chinese Academy of Agricultural Sciences (Beijing, China), and seeds of barley (*Hordeum vulgare* cv. E9) were purchased from the Seed Market of Huazhong Agricultural University (Wuhan, China). One-month-old rice seedlings and one-week-old barley seedlings were used for virulence assays. Seedlings were inoculated with conidial suspensions at 2 × 10^5^ conidia/ml for rice or 3 × 10^4^ conidia/ml for barley in 0.025% Tween-20. Following inoculation, plants were maintained under high humidity at 28°C in darkness the first 36 hpi, then under light. Lesions were photographed and measured at 5 dpi.

To evaluate lesion expansion, rice leaf surfaces were wounded using a punch, and 10 μl of conidial suspension (2 × 10^5^ conidia/ml in 0.025% Tween-20) was applied to each wound. Incubation conditions were as described above. Lesions were imaged and measured at 5 dpi.

For microscopic observation of the infection process, conidial suspensions (2 × 10^5^ conidia/ml) from different strains were applied to the lower epidermis of barley leaves. Leaves were then incubated in a dark, humid chamber at 28°C. The infection process was examined at 24 and 30 hpi using a Nikon Ni90 microscope.

### Glycogen and lipid utilization assay

To evaluate the effects of gene knockout on glycogen and lipid body content, we performed staining to track their dynamics during conidial germination and appressorium formation. Conidia were harvested in sterile ddH_2_O, resuspended to 1 × 10^5^ conidia/mL, and inoculated onto hydrophobic plastic coverslips within a petri dish. Cultures were maintained in a humidified, dark environment. At various time points, samples were collected for staining and microscopic examination. Glycogen was visualized with a KI/I_2_ stain (60 mg/mL potassium iodide, 10 mg/mL iodine), and lipid bodies were detected using a Nile Red stain (Sigma-Aldrich, St. Louis, MO, USA; 50 mM Tris/maleate buffer, 20 mg/mL polyvinylpyrrolidone, 2.5 μg/mL Nile Red, adjusted to pH 7.5). Stained samples were examined by microscopy.

### Mitochondrial staining and observation

To examine the mitochondria across various structures of *M. oryzae*, we used MitoTracker™ Deep Red FM (Thermo Fisher Scientific; catalog number M22426). The dye was diluted 1:500 in sterile ddH_2_O. Samples was incubated on microscope slide in darkness for 5 minutes, then covered with a cover slip, inverted, and mounted on the microscope stage. Mitochondria were observed by confocal microscope in the red spectrum.

### Quantitative PCR (qPCR) analysis

To understand molecular changes during host-pathogen interaction, we extracted total RNA from rice tissues at various time points post-inoculation and performed qPCR to quantify the expression levels of specific genes.

### Protein extraction and western blot analysis

For protein extraction, mycelia cultured in liquid CM for 36 hours were harvested, ground in liquid nitrogen, and resuspended in a protein extraction buffer supplemented with 1 mM PMSF (Sigma-Aldrich, St. Louis, MO, USA). To determine the protein levels of Mrm1 and other mitochondrial proteins, various primary antibodies were applied at 1:5000 dilution (Abmart, Xuhui, Shanghai, China). Immunoreactivity was detected using a compatible secondary antibody at 1:10,000 dilution.

### Phylogenetic analysis

Phylogenetic analysis of Mrm1 orthologs was performed as follows: Full-length protein sequences were aligned using MAFFT v7.490 with the L-INS-i alignment strategy. Poorly conserved gapped regions were trimmed with TrimAl v1.4.1 using a gap threshold of 0.1. Maximum-likelihood phylogenetic trees were constructed in MEGA11 under the Jones-Taylor-Thornton (JTT) amino acid substitution model with gamma-distributed among-site rate variation. Node support was assessed via 1000 bootstrap replicates.

### Polysome profiling

We performed polysome profiling to assess the translational activity and compare overall translational capacity between the wild-type and Δ*mrm1* strains. Mycelia were harvested after 36 hours in liquid CM by vacuum filtration, ground, and lysed in buffer containing Tris-HCl, KCl, MgCl_2_, CHX, BME, DTT, and RNase inhibitor. RNA concentration was determined using a Nanodrop spectrometer, and a portion of the lysate reserved as the input control. Sucrose gradients (15% and 55%) were prepared in polysome buffer using a BioComp Gradient Master. Lysate was loaded onto gradients and centrifuged at 4°C to separate the polysomes. Fractions were collected using a Gradient Station with UV detection at 254 nm. Equal total RNA mass, quantified by Nanodrop A260 absorbance, was loaded from wild-type and Δ*mrm1* lysates to eliminate loading bias. All polysome-to-monosome area ratios were normalized against the corresponding input sample absorbance. Prior to gradient separation, total RNA integrity was verified by agarose gel electrophoresis; no obvious degradation was observed in either strain, ruling out differential RNA breakdown as a confounding variable.

### Subcellular localization analysis

We determined the subcellular localization of Mrm1 by fusing it with a green fluorescent protein (GFP) tag and observing its distribution pattern within fungal cells under confocal microscopy, revealing the potential sites of action within the cell.

## Statistical analysis

Data from multiple trials were averaged, and standard errors were calculated to quantify variability. A t-test was applied for comparing two groups, and ANOVA was used for more than two groups. Significance was determined at *p* < 0.05 or *p* < 0.01, with adjustments for multiple comparisons. Results were considered significantly different at *p* < 0.05 (*) or *p* < 0.01 (**). All raw data and statistical analysis files supporting the results supporting Figures 1–8 and Supplementary Figures S1-S4 have been deposited in ScienceDB (https://www.scidb.cn/) and are publicly available under DOI: [https://doi.org/10.57760/sciencedb.33449] [24].

## Supplementary Material

Supplementary Material.docx

Fig 8F original figure.tif

## Data Availability

The data supporting the findings of this study are available in ScienceDB https://doi.org/10.57760/sciencedb.33449 [24]. Strains and plasmids are available from the corresponding author upon reasonable request.

## References

[1] Asibi AE, Chai Q, Coulter JA. Rice blast: a disease with implications for global food security. Agronomy. 2019;9(8):451. doi: 10.3390/agronomy9080451

[2] Valent B. The impact of blast disease: past, present, and future. Methods Mol Biol. 2021;2356:1–17. 34236673 10.1007/978-1-0716-1613-0_1

[3] Wilson RA, Talbot NJ. Under pressure: investigating the biology of plant infection by *Magnaporthe oryzae*. Nat Rev Microbiol. 2009;7(3):185–195. doi: 10.1038/nrmicro2032 19219052

[4] Eseola AB, Ryder LS, Osés-Ruiz M, et al. Investigating the cell and developmental biology of plant infection by the rice blast fungus *Magnaporthe oryzae*. Fungal Genet Biol. 2021;154:103562. doi: 10.1016/j.fgb.2021.103562 33882359

[5] Kou Y, He Y, Qiu J, et al. Mitochondrial dynamics and mitophagy are necessary for proper invasive growth in rice blast. Mol Plant Pathol. 2019;20(8):1147–1162. doi: 10.1111/mpp.12822 31218796 PMC6640187

[6] Wu S, Zhang Y, Xu L, et al. Mitochondrial outer membrane translocase MoTom20 modulates mitochondrial morphology and is important for infectious growth of the rice blast fungus *Magnaporthe oryzae*. Mol Plant Microbe Interact. 2024;37(4):407–415. doi: 10.1094/MPMI-10-23-0168-R 38171376

[7] Zhong K, Li X, Le X, et al. MoDnm1 dynamin mediating peroxisomal and mitochondrial fission in complex with MoFis1 and MoMdv1 is important for development of functional appressorium in *Magnaporthe oryzae*. PLoS Pathog. 2016;12(8):e1005823. doi: 10.1371/journal.ppat.1005823 27556292 PMC4996533

[8] Cui L, Zheng J, Lin Y, et al. Decoding the ribosome’s hidden language: rRNA modifications as key players in cancer dynamics and targeted therapies. Clin Transl Med. 2024;14(5):e1705. 38797935 10.1002/ctm2.1705 PMC11128715

[9] Jansson MD, Häfner SJ, Altinel K, et al. Regulation of translation by site-specific ribosomal RNA methylation. Nat Struct Mol Biol. 2021;28(11):889–899. doi: 10.1038/s41594-021-00669-4 34759377

[10] Zhou KI, Pecot CV, Holley CL. 2′- O -methylation (nm) in RNA: progress, challenges, and future directions. RNA. 2024;30(5):570–582. doi: 10.1261/rna.079970.124 38531653 PMC11019748

[11] Elliott BA, Ho HT, Ranganathan SV, et al. Modification of messenger RNA by 2′-O-methylation regulates gene expression in vivo. Nat Commun. 2019;10(1):3401. doi: 10.1038/s41467-019-11375-7 31363086 PMC6667457

[12] Li Y, Yi Y, Gao X, et al. 2′-O-methylation at internal sites on mRNA promotes mRNA stability. Mol Cell. 2024;84(12):2320–2336.e6. doi: 10.1016/j.molcel.2024.04.011 38906115 PMC11196006

[13] Barros-Silva D, Klavert J, Jenster G, et al. The role of OncoSnoRNAs and ribosomal RNA 2’-O-methylation in cancer. RNA Biol. 2021;18(sup1):61–74. 34775914 10.1080/15476286.2021.1991167 PMC8677010

[14] Ren Z, Tang B, Xing J, et al. MTA1-mediated RNA m^6^A modification regulates autophagy and is required for infection of the rice blast fungus. New Phytol. 2022;235(1):247–262. doi: 10.1111/nph.18117 35338654

[15] Xie L, Ren S, Zhang L, et al. Mitochondrial translation elongation controls OXPHOS biogenesis by coordinating synthesis and folding of mitochondrially encoded proteins. Mol Cell. 2026;86(8):1511–1528.e12. doi: 10.1016/j.molcel.2026.03.017 41997111

[16] Helm M, Motorin Y. Detecting RNA modifications in the epitranscriptome: predict and validate. Nat Rev Genet. 2017;18(5):275–291. doi: 10.1038/nrg.2016.169 28216634

[17] Roundtree IA, Evans ME, Pan T, et al. Dynamic RNA modifications in gene expression regulation. Cell. 2017;169(7):1187–1200. doi: 10.1016/j.cell.2017.05.045 28622506 PMC5657247

[18] Sharma S, Lafontaine DLJ. ‘View from a bridge’: a new perspective on eukaryotic rRNA base modification. Trends Biochem Sci. 2015;40(10):560–575. 26410597 10.1016/j.tibs.2015.07.008

[19] Watkins NJ, Bohnsack MT. The box C/D and H/ACA snoRnps: key players in the modification, processing and the dynamic folding of ribosomal RNA. Wiley Interdiscip Rev RNA. 2012;3(3):397–414. doi: 10.1002/wrna.117 22065625

[20] Rebelo-Guiomar P, Pellegrino S, Dent KC, et al. A late-stage assembly checkpoint of the human mitochondrial ribosome large subunit. Nat Commun. 2022;13(1):929. doi: 10.1038/s41467-022-28503-5 35177605 PMC8854578

[21] Sirum-Connolly K, Peltier JM, Crain PF, et al. Implications of a functional large ribosomal RNA with only three modified nucleotides. Biochimie. 1995;77(1–2):30–39. doi: 10.1016/0300-9084(96)88101-6 7541254

[22] Lövgren JM, Wikström PM. The rlmB gene is essential for formation of Gm2251 in 23S rRNA but not for ribosome maturation in *Escherichia coli*. J Bacteriol. 2001;183(23):6957–6960. 11698387 10.1128/JB.183.23.6957-6960.2001 PMC95539

[23] Peng YL, Shishiyama J. Temporal sequence of cytological events in rice leaves infected with *pyricularia oryzae*. Can J Bot. 1988;66(4):730–735. doi: 10.1139/b88-107

[24] Chen X. The original experimental data of the manuscript. Sci Data Bank. 2025. doi: 10.57760/sciencedb.33449

