# Supplementary material for: A putative rRNA methyltransferase Mrm1 regulates mitochondrial dynamics and pathogenicity in Magnaporthe oryzae
Source: Virulence. 2026 Aug 12;17(1):2715237. doi: 10.1080/21505594.2026.2715237 (PMC13471095; doi:10.1080/21505594.2026.2715237)
Supplement: Supplementary file 1 [file KVIR_A_2715237_SM5924.docx]

**Supplemental data:** Mi *et al.* (2026). A putative rRNA methyltransferase Mrm1 regulates mitochondrial dynamics and pathogenicity in *Magnaporthe oryzae*

**Fig. S1** Expression level of *MRM1* gene at different development stages of *M. oryzae*.

HY, Mycelial hyphae; CO, Conidia; AP_3H, Appressoria at 3 hpi; AP_12H, Appressoria at 12 hpi; IH_18H, Invasive hyphae at 18 hpi; IH_24H, Invasive hyphae at 24 hpi; IH_48H, Invasive hyphae at 48 hpi.

**Fig. S2** *MRM1* gene disruption via split-PCR strategy and PCR validation of transformants.

**A** The upstream and downstream flanking regions of 2 kb around the *MRM1* gene were amplified. The Split-PCR method was utilized to fuse the 1.5 kb segments from both sides with the HYG gene sequence, yielding the recombinant fragment. **B** PCR was conducted to check for the integration and deletion of the *MRM1* gene and its 2 kb flanking sequences. LB: Verification of the left arm of the *MRM1* gene 2 kb using primer pair LCK and HCK-UP; RB: Verification of the right arm of the MRM1 gene using primer pair RCK and HCK-DOWN; H_2_O denotes the negative control, while WT denotes the wild-type positive control.

**Fig. S3** Comparative analysis of vegetative growth and hyphal tip staining in wild-type and Δ*mrm1* strains of *M. oryzae*.

**A** Growth rate assessment of the wild-type and Δ*mrm1* strains following 120 hpi. **B** Measurement of colony diameter, with significant differences from the wild-type indicated by asterisks (*, P < 0.05). **C** Calcofluor White (CFW) staining of hyphal tips to visualize septum distribution, with white arrows marking the cell septa. Scale bar, 20 μm. **D** Quantification of average hyphal tip cell length, highlighting significant differences from the wild-type with an asterisk (**, P < 0.01).

**Fig. S4** Observation of conidium and spore morphology in wild-type and Δ*mrm1* strains.

**A** Examination of conidiophore morphology. **B** Statistics of sporulation, with significant differences from the wild type indicated by asterisks (**, P < 0.01). **C** Analysis of conidial morphology. **D** Distribution of different conidial morphologies, highlighting significant differences from the wild type with asterisks (**, P < 0.01). Scale bar, 50 μm.

**Table S1 Fungal strains used in this study.**

| **Strains** | **Genotypes** | **References** |
| --- | --- | --- |
| P131 | A wild-type isolate of *M. oryzae* | This study |
| Δ*mrm1-1* | *MRM1* deletion mutants of P131. | This study |
| Δ*mrm1-2* | *MRM1* deletion mutants of P131. | This study |
| Δ*mrm1/MRM1* | Δ*mrm1* transformed by *M. oryzae MRM1 gene*. | This study |
| WT/GFP-Mrm1 | P131 transformed by *eGFP-MRM1* fusion construct. | This study |
| Δ*mrm1/GFP-Mrm1* | Δ*mrm1* transformed by *eGFP-MRM1* fusion construct. | This study |
| Δ*mrm1/MRM1^ND^-1* | Δ*mrm1* transformed by *eGFP-MRM1*^ND^ fusion construct. | This study |
| Δ*mrm1/MRM1^ND^-2* | Δ*mrm1* transformed by *eGFP-MRM1*^ND^ fusion construct. | This study |
| Δ*mrm1/MRM1^MD^-1* | Δ*mrm1* transformed by *eGFP-MRM1*^MD^ fusion construct. | This study |
| Δ*mrm1/MRM1^MD^-2* | Δ*mrm1* transformed by *eGFP-MRM1*^MD^ fusion construct. | This study |
| Δ*mrm1/MRM1^MTS^-7* | Δ*mrm1* transformed by *eGFP-MRM1*^MTS^ fusion construct. | This study |
| Δ*mrm1/MRM1^MTS^-10* | Δ*mrm1* transformed by *eGFP-MRM1*^MTS^ fusion construct. | This study |
| Δ*mrm1/NcMRM1-18* | Δ*mrm1* transformed by *N. crassa* *MRM1 gene*. | This study |
| Δ*mrm1/NcMRM1-19* | Δ*mrm1* transformed by *N. crassa* *MRM1 gene*. | This study |
| Δ*mrm1/NcMRM1-20* | Δ*mrm1* transformed by *N. crassa* *MRM1 gene*. | This study |

Table S2 Primers that used in this study.

| Primers | Sequences (5'–3') |
| --- | --- |
| HPT-LCK | GACAGACGTCGCGGTGAGTT |
| HPT-RCK | TCTGGACCGATGGCTGTGTAG |
| HPT-F1 | CTCCGACCTGATGCAGCTCT |
| HPT-R1 | CTCGCTCCAGTCAATGACC |
| HYG-LB | ACCTCCACTAGCTCCAGCCAAG |
| HYG-RB | GAATAGAGTAGATGCCGACCGGG |
| PAL1LBCK | GATGGCCTCCTGTCTACTACGT |
| PAL1LB-F | TGATGGCCTCCTGTCTACTA |
| PAL1LB-R | ACCTCCACTAGCTCCAGCCAAGAGATCTTGCT CCGCAAAA |
| PAL1RB-F | GAATAGAGTAGATGCCGACCGGGACTGACAAAGCATTC |
| PAL1RB-R | GCTGCGAGATCTTGCCAC |
| PAL1RBCK | TTGAAGAGTCCCACAGTCCA |
| PAL1-F  PAL1-R | ATGTCATCGG GAATGCATCC  GGATGCCTCCGCTCGAAGTA |
| PAL1GFP-F | GAGGTCGACGGTATCGATAAGCTTGTATTGACACGCATTGAGAC |
| PAL1GFP-R | AGCACCTCTAGAACTAGTGGATCCGGCTGCGGTACCCGGGGCCG |
| SLA1RFP-F | GGTATCGATAAGCTTATGGGCTTCATCGGCGTCTA |
| SLA1RFP-R | AATCCTTCTTGGATCCTTAGAAGCCAAATGGATTCT |
| ATG8-GFP-F | ATGGACGAGCTGTACAAGGGATCCATGCGCTCCAAGTTCAAGGA |
| ATG8-GFP-R | ATGGACGAGCTGTACAAGGGATCCATGCGCTCCAAGTTCAAGGA |
| Lifeact-RFP-F | TTTCTCGAGATGGCATCCACGGGCGTC |
| Lifeact-RFP-R | TTTCTCGAGATGGCATCCACGGGCGTC |
| SEP5GFP-F | TTCGAATTCCAAATTGTGAAATTGCAT |
| SEP5GFP-R | TTTGGATCCCTCGGTAGTAGTGGTTTC |
| SEP6GFP-F | TTCAAGCTTGGGTTGGTGGGTGGGTGC |
| SEP6GFP-R | TTAGGATCCACCCGAAACTACCATTAAAC |
| PAL1PKN-F | GTCGACGGTATCGATAAGCT TGTATTGACACGCATTGAGAC |
| PAL1PKN-R | TAATCTAGAACTAGTGGATCCATGACGAATGTAGCGCATTA |
| SLA1PGPBKT7-F | ATGGCCATGGAGGCCGAATTCATGGGCTTCATCGGCGTCTA |
| SLA1PGPBKT7-R | GGCCGCTGCAGGTCGACGGATCCTTAGAAGCCAAATGGATTCT |
| PAL1PGADT7-F | GGTGGGCATCGATACGGGATCCATGTCATCGGGAATGCATCC |
| PAL1PGADT7-R | TGCAGCTCGAGCTCGATGGATCCCTAGGCTGCGGTACCCGGGG |

Table S3 Plasmids used in this study.

| Names | Descriptions |
| --- | --- |
| pKN | Vector used to construct complementation vectors and other vectors; with the *NPTII* gene as a selective marker inserted into pKS^+^ (Yang et al., 2010). |
| pKN-*PAL1* | *PAL1* complementation vector; *PAL1* gene containing 1.5 kb promoter and 0.5 kb terminator regions were amplified and inserted into pKN. |
| pKNRG | Vector used to construct vectors to constitutively express selected genes; with the fungal constitutive promoter RP27 (Yang et al., 2010). |
| pKNRG-*PAL1* | Vector for sub-cellular localization of PAL1 protein; coding region of *PAL1* was cloned into vector pKNRG. |
| pKNRG-*Rab5* | Vector for sub-cellular localization of Rab5 protein; coding region of *Rab5* was cloned into vector pKNRG. |
| pKNRG-*SEP5* | Vector for sub-cellular localization of SEP5 protein; coding region of *SEP5* was cloned into vector pKNRG. |
| pKNRG-*SEP6* | Vector for sub-cellular localization of SEP6 protein; coding region of *SEP6* was cloned into vector pKNRG. |
| pKNRR-*SLA1* | Vector for sub-cellular localization of SLA1 protein; coding region of *SLA1* was cloned into vector pKNRR. |
| pKNRR-LifeAct | Vector for sub-cellular localization of LifeAct protein; coding region of *LifeAct* was cloned into vector pKNRR. |
| pKNRG-*ATG8* | Vector for sub-cellular localization of ATG8 protein; coding region of *ATG8* was cloned into vector pKNRG. |
